# Dissipative generation of significant amount of mechanical entanglement in a coupled optomechanical system

**DOI:** 10.1038/s41598-017-15032-1

**Published:** 2017-11-03

**Authors:** Rong-Xin Chen, Chang-Geng Liao, Xiu-Min Lin

**Affiliations:** 1 Institute for Quantum Science and Engineering, Texas A&M University, College Station, TX 77843 USA; 2 Fujian Provincial Key Laboratory of Quantum Manipulation and New Energy Materials, College of Physics and Energy, Fujian Normal University, Fuzhou, 350117 China; 3 Fujian Provincial Collaborative Innovation Center for Optoelectronic Semiconductors and Efficient Devices, Xiamen, 361005 China; 4 Department of Electronic Engineering, Fujian Polytechnic of Information Technology, Fuzhou, 350003 China

## Abstract

We propose an approach for generating steady-state mechanical entanglement in a coupled optomechanical system. By applying four-tone driving lasers with weighted amplitudes and specific frequencies, we obtain an effective Hamiltonian that couples the delocalized Bogoliubov modes of the two mechanical oscillators to the cavity modes via beam-splitter-like interactions. When the mechanical decay rate is small, the Bogoliubov modes can be effectively cooled by the dissipative dynamics of the cavity modes, generating steady-state entanglement of the mechanical modes. The mechanical entanglement obtained in the stationary regime is strongly dependent on the values of the ratio of the effective optomechanical coupling strengths. Numerical simulation with the full linearized Hamiltonian shows that significant amount of mechanical entanglement can indeed be obtained by balancing the opposing effects of varying the ratio and by carefully avoiding the system parameters that may lead to amplified oscillations of the mechanical mean values detrimental to the entanglement generation.

## Introduction

Entanglement, especially the entanglement of macroscopic objects, is of great interest both for fundamental physics and for possible applications in quantum information processing. Recent experimental progress makes it possible to manipulate quantum states of macroscopic mechanical objects by means of optical or microwave radiation pressure. Many schemes have been proposed to achieve mechanical entanglement, strong electromechanical coupling, and quantum state transfer in opto-and electro-mechanical systems^1–7^. In particular, one can enhance the entanglement between a mechanical resonator and a cavity field by applying suitable time modulation of the driving lasers^1,2^. By using an auxiliary mode in a three-mode system, an effective two-mode-squeezing interaction between two target modes for acquiring optomechanical entanglement can be achieved^8–11^. The construction of three-mode optomechanical system has recently been widely studied^12–17^ and realized experimentally^18–20^. Nevertheless, the amount of optomechanical entanglement achieved in these schemes based on the coherent parametric interactions is generally subjected to an upper bound imposed by the stability constraint of the systems^21^. The idea of reservoir engineering, which is previously proposed to cool the trapped ions by lasers^22^, is found to be useful for obtaining large entanglement in the context of atomic systems^23–27^ and has even been realized in experiments^28^. Recently, the reservoir-engineering-based mechanism is exploited in optomechanical systems to create significant amount of steady-state mechanical squeezing^29^, cavity-cavity entanglement^30,31^, and cavity-mechanical entanglement^15,32–35^ which can largely surpass the upper bound^21^.

In this work, we consider the generation of entanglement between two remote mechanical oscillators in a coupled optomechanical system using the reservoir-engineering-based mechanism^29–33^. We note that the generation of distant mechanical entanglement in coupled optomechanical systems can be achieved via optical-fiber mediated coupling^36^ and by periodically modulating the pumping amplitudes^37,38^. Here, based on the reservoir-engineering method, the created steady-state mechanical entanglement is significantly larger than that obtained in refs^36,37^, and requires only one, rather than two^38^, steps of implementing the driving lasers. The key for greatly enhancing the entanglement is to drive the coupled cavity modes with four-tone lasers of weighted amplitudes and specific frequencies so that we obtain an effective system Hamiltonian where two nonlocal Bogoliubov modes of the mechanical oscillators are coupled to the cavity modes via beam-splitter-like interactions. Notably, the Bogoliubov modes can be sufficiently cooled via swapping quanta with the cavity modes which interact with optical thermal baths with neglectful mean photons. In this way, after some time of dissipative dynamics, the mechanical modes are driven to close to a two-mode squeezed state which is, in fact, the joint vacuum of two Bogoliubov modes being cooled. The amount of entanglement is independent of initial states but is strongly dependent on the ratio of the effective optomechanical couplings rather than simply on their magnitudes. The change in the ratio will simultaneously have two confronting effects on creating mechanical entanglement. The entanglement can be maximized by balancing the confronting effects through choosing proper asymmetric driving amplitudes and as small mechanical decay rate as possible. Our numerical results with the full linearized Hamiltonian show that significant amount of mechanical entanglement can indeed be generated. In particular, we observe obvious amplified self-sustained oscillations of mechanical mean values for some system parameters, which may result from the intrinsic nonlinearity of the optomechanial interaction. The effects of these amplified oscillations in some parameter regimes, often unwanted in the generation of mechanical entanglement and largely unconsidered in many optomechanical schemes, have been numerically analyzed and been carefully avoided in our scheme.

## Model and quantum Langevin equations

**Figure 1:**
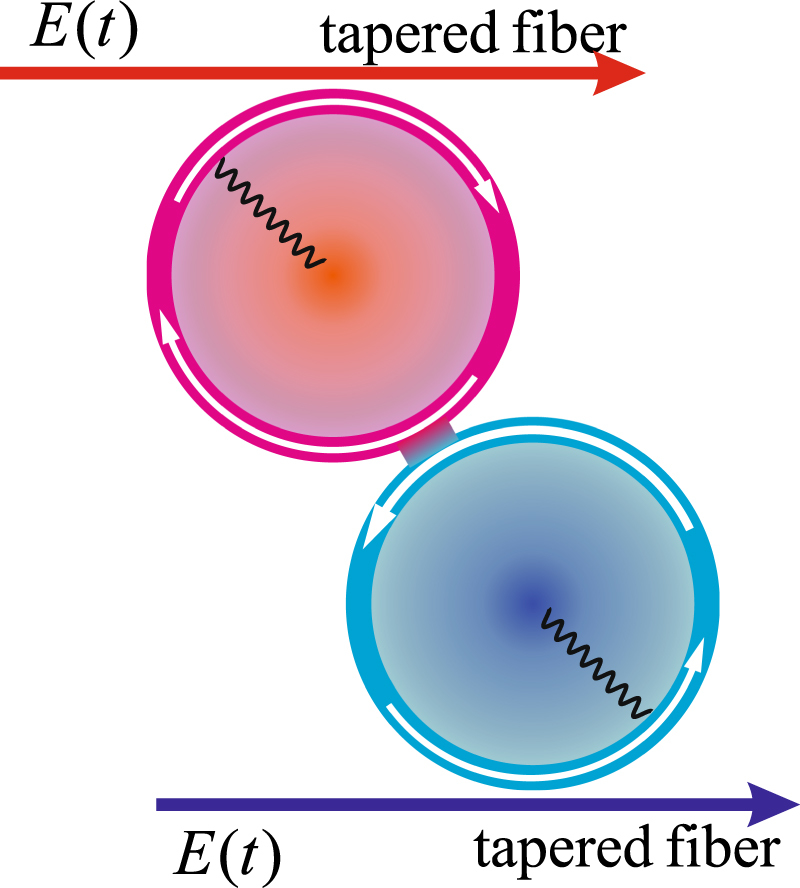
Schematic representation of coupled microtoroidal resonators with a tunneling coupling. Each resonator is coupled to a mechanical mode and pumped by the tapered fibers.

As illustrated in Fig. 1, we consider a coupled microtoroidal optomechanical system^39–43^ where two phonon modes *B*_1_ and *B*_2_ respectively interact with two photon modes *A*_1_ and *A*_2_ which in turn are coupled via the photon tunneling. The arrangement is assumed to be symmetrical, i.e., the two mechanical oscillators have the same frequency *ω*_*m*_ and damping rate *γ*_*m*_. The cavity modes with frequency *ω*_*c*_ are driven by lasers with the frequency *ω*_*L*_ and time-modulated amplitude *E*(*t*) through the tapered fibers. In the rotating frame with respect to laser frequency *ω*_*L*_, the Hamiltonian of our system reads (*ħ* = 1)

1 $$H=\sum _{j=1,2}\,[{{\rm{\Delta }}}_{0}{A}_{j}^{\dagger }{A}_{j}+{\omega }_{m}{B}_{j}^{\dagger }{B}_{j}-g{A}_{j}^{\dagger }{A}_{j}({B}_{j}+{B}_{j}^{\dagger })+iE(t){A}_{j}^{\dagger }-iE{(t)}^{\ast }{A}_{j}]+J({A}_{1}{A}_{2}^{\dagger }+{A}_{1}^{\dagger }{A}_{2}),$$

where *A*_*j*_ (${A}_{j}^{\dagger }$) and *B*_*j*_ (${B}_{j}^{\dagger }$) are the annihilation (creation) operators of the *j*th photon mode and phonon mode, respectively. Δ_0_ = *ω*_*c*_ − *ω*_*L*_ denotes the detuning between the cavity and the driving field. The parameters *g* and *J* represent the strengths of the single-photon optomechanical interaction and photon tunneling, respectively.

The dynamics of our system can be described by a set of quantum Langevin equations (QLEs)^44^:

2a $${\dot{A}}_{1}=-(\kappa /2+i{{\rm{\Delta }}}_{0}){A}_{1}-iJ{A}_{2}+ig{A}_{1}({B}_{1}+{B}_{1}^{\dagger })+E(t)+\sqrt{\kappa }{a}_{1}^{in}(t),$$

2b $${\dot{A}}_{2}=-(\kappa /2+i{{\rm{\Delta }}}_{0}){A}_{2}-iJ{A}_{1}+ig{A}_{2}({B}_{2}+{B}_{2}^{\dagger })+E(t)+\sqrt{\kappa }{a}_{2}^{in}(t),$$

2c $${\dot{B}}_{j}=-({\gamma }_{m}/2+i{\omega }_{m}){B}_{j}+ig{A}_{j}^{\dagger }{A}_{j}+\sqrt{{\gamma }_{m}}{b}_{j}^{in}(t\mathrm{)}.$$

Here, *κ* is the cavity decay rate; ${a}_{j}^{in}(t)$ and ${b}_{j}^{in}(t)$ stand for independent input vacuum noise operators with zero mean value and the following nonzero auto-correlation functions:

3a $$\langle {a}_{j}^{in}(t){a}_{j}^{in\dagger }(t^{\prime} )\rangle =\delta (t-t^{\prime} ),$$

3b $$\langle {b}_{j}^{in}(t){b}_{j}^{in\dagger }(t^{\prime} )\rangle =({\bar{n}}_{b}+\mathrm{1)}\,\delta (t-t^{\prime} ),$$

3c $$\langle {b}_{j}^{in\dagger }(t){b}_{j}^{in}(t^{\prime} )\rangle ={\bar{n}}_{b}\delta (t-t^{\prime} ),$$

where ${\bar{n}}_{b}$ is the mean thermal occupancy of the mechanical baths.

In the presence of strong external pumping, we can write the system operators as *A*_*j*_ = *α*_*j*_(*t*) + *a*_*j*_ and *B*_*j*_ = *β*_*j*_(*t*) + *b*_*j*_ where *a*_*j*_ and *b*_*j*_ are quantum fluctuation operators with zero mean value around classical *c*-number amplitudes *α*_*j*_(*t*) and *β*_*j*_(*t*) of the system operators, respectively. Under the conditions |*α*_*j*_(*t*)|, $|{\beta }_{j}(t)|\gg 1$, standard linearization techniques^4^ can be applied by substituting *A*_*j*_ = *α*_*j*_(*t*) + *a*_*j*_ and *B*_*j*_ = *β*_*j*_(*t*) + *b*_*j*_ into Eq. (2). In this way, we obtain a set of nonlinear differential equations for the classical mean values *α*_*j*_(*t*) and *β*_*j*_(*t*) only (discarding the terms with quantum fluctuation operators and quantum noise operators)

4a $$\dot{\alpha }(t)=[-\kappa /2+i({{\rm{\Delta }}}_{0}+J)]\alpha (t)+ig\alpha (t)\,[\beta (t)+\beta {(t)}^{\ast }]+E(t),$$

4b $$\dot{\beta }(t)=-({\gamma }_{m}/2+i{\omega }_{m})\,\beta (t)+ig{|\alpha (t)|}^{2},$$

where we have assumed *α*_1_(*t*) = *α*_2_(*t*) = *α*(*t*) and *β*_1_(*t*) = *β*_2_(*t*) = *β*(*t*) considering the system symmetry. One can also get the following linearized QLEs for the quantum fluctuations by neglecting the terms containing classical mean values only and all nonlinear terms such as *a*_1_ *b*_1_ and ${a}_{2}{b}_{2}^{\dagger }$

5a $${\dot{a}}_{1}=(-\kappa /2+i{{\rm{\Delta }}}_{0}){a}_{1}-iJ{a}_{2}+ig\{{a}_{1}[\beta (t)+\beta {(t)}^{\ast }]+\alpha (t)\,({b}_{1}+{b}_{1}^{\dagger })\}+\sqrt{\kappa }{a}_{1}^{in}(t),$$

5b $${\dot{a}}_{2}=(-\kappa /2+i{{\rm{\Delta }}}_{0}){a}_{2}-iJ{a}_{1}+ig\{{a}_{2}[\beta (t)+\beta {(t)}^{\ast }]+\alpha (t)\,({b}_{2}+{b}_{2}^{\dagger })\}+\sqrt{\kappa }{a}_{2}^{in}(t),$$

5c $${\dot{b}}_{j}=(-{\gamma }_{m}/2+i{\omega }_{m}){b}_{j}+ig[{a}_{j}^{\dagger }\alpha (t)+{a}_{j}\alpha {(t)}^{\ast }]+\sqrt{{\gamma }_{m}}{b}_{j}^{in}(t),$$

which correspond to a system Hamiltonian with linearized optomechanical interactions

6 $${H}^{{\rm{lin}}}=\sum _{j=1,2}\,\{{\rm{\Delta }}(t){a}_{j}^{\dagger }{a}_{j}+{\omega }_{m}{b}_{j}^{\dagger }{b}_{j}+[G{(t)}^{\ast }{a}_{j}+G(t){a}_{j}^{\dagger }]\,({b}_{j}^{\dagger }+{b}_{j})\}+J({a}_{1}^{\dagger }{a}_{2}+{a}_{2}^{\dagger }{a}_{1}),$$

with Δ(*t*) = Δ_0_ − *g*[*β*(*t*) + *β*(*t*)^*^] and *G*(*t*) = −*gα*(*t*) being the effective detuning and enhanced optomechanical coupling, respectively.

## Effective Hamiltonian and the mechanism

In this paper, we focus on the weak optomechanical coupling regime, namely $|g/{\omega }_{m}|\ll 1$. In this case, approximate analytical solutions for Eq. (4) can be found by expanding the classical mean values *α*(*t*) and *β*(*t*) in powers of *g* as^29,30,38^

7a $$\alpha (t)=\alpha {(t)}^{\mathrm{(0)}}+\alpha {(t)}^{\mathrm{(1)}}+\alpha {(t)}^{\mathrm{(2)}}+\cdots ,$$

7b $$\beta (t)=\beta {(t)}^{\mathrm{(0)}}+\beta {(t)}^{\mathrm{(1)}}+\beta {(t)}^{\mathrm{(2)}}+\cdots .$$

Substituting these expressions into Eq. (4), one finds the equations for zero order of *g*

8a $${\dot{\alpha }}^{\mathrm{(0)}}=[-\kappa /2+i({{\rm{\Delta }}}_{0}+J)]{\alpha }^{\mathrm{(0)}}+E(t),$$

8b $${\dot{\beta }}^{\mathrm{(0)}}=(-{\gamma }_{m}/2+i{\omega }_{m}){\beta }^{\mathrm{(0)}},$$

When a four-tone driving laser $E(t)={\sum }_{k=1}^{4}{E}_{k}{e}^{-i{\omega }_{k}t}$ is implemented, the asymptotic solutions for time $t\gg 1/\kappa$, 1/*γ*_*m*_ are given by

9 $$\alpha {(t)}^{\mathrm{(0)}}=\sum _{k=1}^{4}\,{\overline{\alpha }}_{k}{e}^{-i{\omega }_{k}t},\beta {(t)}^{\mathrm{(0)}}=\mathrm{0,}$$

where

10 $${\overline{\alpha }}_{k}={E}_{k}/[\kappa /2+i({{\rm{\Delta }}}_{0}+J-{\omega }_{k})],$$

One can follow similar procedures to derive higher-order corrections which are tedious and will not be presented here. In view of *α*(*t*)^(1)^ = 0, $|\alpha {(t)}^{\mathrm{(2)}}|\ll |\alpha {(t)}^{\mathrm{(0)}}|$, and $|g[\beta (t)+{\beta }^{\ast }(t)]|\ll {{\rm{\Delta }}}_{0}\sim {\omega }_{m}$, we can make the approximations $\alpha (t)\simeq \alpha {(t)}^{\mathrm{(0)}}$ and ${\rm{\Delta }}(t)\simeq {{\rm{\Delta }}}_{0}$. In the asymptotic regime, the Hamiltonian in Eq. (6) then becomes

11 $${H}_{asy}^{lin}=\sum _{j=1,2}\{{{\rm{\Delta }}}_{0}{a}_{j}^{\dagger }{a}_{j}+{\omega }_{m}{b}_{j}^{\dagger }{b}_{j}+[\bar{G}{(t)}^{\ast }{a}_{j}+\bar{G}(t){a}_{j}^{\dagger }]\,({b}_{j}^{\dagger }+{b}_{j})\}\,+J({a}_{1}^{\dagger }{a}_{2}+{a}_{2}^{\dagger }{a}_{1}),$$

where

12 $$\overline{G}(t)=-g\alpha {(t)}^{\mathrm{(0)}}=\sum _{g=1}^{4}\,{\overline{G}}_{k}{e}^{-i{\omega }_{k}t},$$

with $${\overline{G}}_{k}=-g{\overline{\alpha }}_{k}$$.

**Figure 2:**
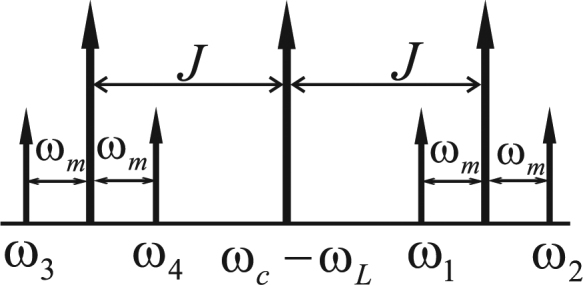
Representation of the modulating frequencies of the four-tone driving pulse.

To obtain the targeted Hamiltonian, we select the modulating frequencies *ω*_*k*_ as specified in Fig. 2. We then rewrite the Eq. (11) in the interaction picture of ${{\rm{\Delta }}}_{0}{a}_{j}^{\dagger }{a}_{j}+{\omega }_{m}{b}_{j}^{\dagger }{b}_{j}$ and make the rotating-wave approximation by neglecting all fast oscillating terms under the conditions *J* > 2*ω*_*m*_ and ${\omega }_{m}\gg {\overline{G}}_{k}$ to arrive at the effective Hamiltonian

13 $$\begin{array}{rll}{H}_{eff} & = & \frac{1}{2}[({\bar{G}}_{1}+{\bar{G}}_{3}){b}_{1}+({\bar{G}}_{1}-{\bar{G}}_{3}){b}_{2}+({\bar{G}}_{2}+{\bar{G}}_{4}){b}_{1}^{\dagger }+({\bar{G}}_{2}-{\bar{G}}_{4}){b}_{2}^{\dagger }]{a}_{1}^{\dagger }\\  &  & +\frac{1}{2}[({\bar{G}}_{1}-{\bar{G}}_{3}){b}_{1}+({\bar{G}}_{1}+{\bar{G}}_{3}){b}_{2}+({\bar{G}}_{2}-{\bar{G}}_{4}){b}_{1}^{\dagger }+({\bar{G}}_{2}+{\bar{G}}_{4}){b}_{2}^{\dagger }]{a}_{2}^{\dagger }+{H}.{c}.\end{array}$$

Assuming that

14 $${\overline{G}}_{2}=-{\overline{G}}_{4}={G}_{+},{\overline{G}}_{1}={\overline{G}}_{3}={G}_{-},|{G}_{-}| > |{G}_{+}|,$$

we finally have the following kind of Hamiltonian with beam-splitter-like interactions

15 $${H}_{eff}=\tilde{G}({\theta }_{1}{a}_{1}^{\dagger }+{\theta }_{2}{a}_{2}^{\dagger })+H.c.,$$

where $\tilde{G}=\sqrt{{G}_{-}^{2}-{G}_{+}^{2}}$. The introduced Bogoliubov modes *θ*_1_ and *θ*_2_ are defined as unitary transformations of the mechanical modes *b*_1_ and *b*_2_ with a two-mode squeezed operator, respectively,

16a $${\theta }_{1}=S(r){b}_{1}{S}^{\dagger }(r)={b}_{1}\,\cosh \,r+{b}_{2}^{\dagger }\,\sinh \,r,$$

16b $${\theta }_{2}=S(r){b}_{2}{S}^{\dagger }(r)={b}_{2}\,\cosh \,r+{b}_{1}^{\dagger }\,\sinh \,r,$$

16c $$S(r)=\exp [r({b}_{1}{b}_{2}-{b}_{1}^{\dagger }{b}_{2}^{\dagger })],$$

16d $$r={\tanh }^{-1}({G}_{+}/{G}_{-}\mathrm{)}.$$

Note that the joint ground state of *θ*_1_ and *θ*_2_ is the two-mode squeezed vacuum state of the mechanical modes *b*_1_ and *b*_2_, which can be readily checked ${\theta }_{j}[S(r)\,{|00\rangle }_{{b}_{1}{b}_{2}}]=S(r){b}_{j}{S}^{\dagger }(r)S(r)\,{|00\rangle }_{{b}_{1}{b}_{2}}=0$.

For sufficiently small mechanical damping rate, the mechanical modes only weakly interact with the mechanical thermal baths with relatively large mean thermal occupancies. The dynamics of mechanical modes, i.e., the Bogoliubov modes, is dominated by the coupling to the cavity modes. In fact, the Bogoliubov modes *θ*_1_ and *θ*_2_ can be simultaneously cooled to near ground states after long enough time via the beam-splitter-like interactions with cavity modes *a*_1_ and *a*_2_ which in turn strongly couple to optical thermal baths with neglectable small thermal occupancies. In this way, the dissipation of the cavity modes is exploited to cool the Bogoliubov modes, in other words, to generate two-mode squeezed (thermal) states of the mechanical modes.

## Evolution equation of the covariance matrix

The fact that the dynamics of our four-mode bosonic system is governed by a linearized Hamiltonian ensures that the evolved states are Gaussian states whose information-related properties are fully represented by the 8 × 8 covariance matrix (CM) *σ* with entries defined as^45–47^

17 $${\sigma }_{j,k}=\langle {R}_{j}{R}_{k}+{R}_{k}{R}_{j}\rangle /2.$$

Here $R={({q}_{{b}_{1}},{p}_{{b}_{1}},{q}_{{b}_{2}},{p}_{{b}_{2}},{q}_{{a}_{1}},{p}_{{a}_{1}},{q}_{{a}_{2}},{p}_{{a}_{2}})}^{T}$ is a vector of dimensionless quadrature operators related to bosonic modes *o* via ${q}_{o}=(o+{o}^{\dagger })/\sqrt{2}$ and ${p}_{o}=(o-{o}^{\dagger })/(i\sqrt{2})$. By further introducing the vector of input noise quadrature operators

18 $$N={(\sqrt{{\gamma }_{m}}{q}_{{b}_{1}^{in}},\sqrt{{\gamma }_{m}}{p}_{{b}_{1}^{in}},\sqrt{{\gamma }_{m}}{q}_{{b}_{2}^{in}},\sqrt{{\gamma }_{m}}{p}_{{b}_{2}^{in}},\sqrt{\kappa }{q}_{{a}_{1}^{in}},\sqrt{\kappa }{p}_{{a}_{1}^{in}},\sqrt{\kappa }{q}_{{a}_{2}^{in}},\sqrt{\kappa }{p}_{{a}_{2}^{in}})}^{T},$$

we can transform the QLEs for the quantum fluctuations in Eq. (5) into a more compact form

19 $$\dot{R}=MR+N.$$

Here *M* is an 8 × 8 real coefficient matrix

20 $$M=(\begin{array}{cccccccc}-{\gamma }_{{\rm{m}}}/2 & {\omega }_{{\rm{m}}} & 0 & 0 & 0 & 0 & 0 & 0\\ -{\omega }_{{\rm{m}}} & -{\gamma }_{{\rm{m}}}/2 & 0 & 0 & -2{G}_{{\rm{R}}}(t) & -2{G}_{{\rm{I}}}(t) & 0 & 0\\ 0 & 0 & -{\gamma }_{{\rm{m}}}/2 & {\omega }_{{\rm{m}}} & 0 & 0 & 0 & 0\\ 0 & 0 & -{\omega }_{{\rm{m}}} & -{\gamma }_{{\rm{m}}}/2 & 0 & 0 & -2{G}_{{\rm{R}}}(t) & -2{G}_{{\rm{I}}}(t)\\ 2{G}_{{\rm{I}}}(t) & 0 & 0 & 0 & -\kappa /2 & {\rm{\Delta }}(t) & 0 & J\\ -2{G}_{{\rm{R}}}(t) & 0 & 0 & 0 & -{\rm{\Delta }}(t) & -\kappa /2 & -J & 0\\ 0 & 0 & 2{G}_{{\rm{I}}}(t) & 0 & 0 & J & -\kappa /2 & {\rm{\Delta }}(t)\\ 0 & 0 & -2{G}_{{\rm{R}}}(t) & 0 & -J & 0 & -{\rm{\Delta }}(t) & -\kappa /2\end{array}),$$

where *G*_*R*_(*t*) and *G*_*I*_(*t*) are respectively real and imaginary parts of the effective coupling *G*(*t*). Given that the quantum states of our system remain Gaussian throughout the evolution, the QLEs in Eq. (5) is equivalent to the equation of motion for the CM. From Eqs (3), (17) and (19), we can deduce a linear differential equation for the CM^1^

21 $$\dot{\sigma }=M\sigma +\sigma {M}^{T}+D,$$

where *D* is a diffusion matrix whose components are associated with the input noise correlation functions in Eq. (3)

22 $${D}_{j,k}\delta (t-t^{\prime} )=\langle {N}_{j}(t){N}_{k}(t^{\prime} )+{N}_{k}(t^{\prime} ){N}_{j}(t)\rangle /2.$$

It is found that *D* is diagonal

23 $$D=\frac{1}{2}\times {\rm{diag}}[{\gamma }_{{\rm{m}}}\mathrm{(2}{\bar{{\rm{n}}}}_{{\rm{b}}}+\mathrm{1),}{\gamma }_{{\rm{m}}}\mathrm{(2}{\bar{{\rm{n}}}}_{{\rm{b}}}+\mathrm{1)},{\gamma }_{{\rm{m}}}\mathrm{(2}{\bar{{\rm{n}}}}_{{\rm{b}}}+1),{\gamma }_{{\rm{m}}}\mathrm{(2}{\bar{{\rm{n}}}}_{{\rm{b}}}+1),\kappa ,\kappa ,\kappa ,\kappa ]$$

In the following, we will utilize the Eq. (21) to study the time evolution of the mechanical entanglement. Note that the coefficient matrices in Eq. (20) correspond to the system Hamiltonian in Eq. (6) where the only approximation is the commonly used linearization techniques in optomechanics.

## Discussion

The entanglement of two mechanical oscillators can be calculated from the two-mode CM *σ*_*m*_ which is the first four rows and columns of the CM *σ* for the whole system. When *σ*_*m*_ is arranged in the following block form

24 $${\sigma }_{m}=(\begin{array}{cc}{V}_{1} & {V}_{3}\\ {V}_{3}^{T} & {V}_{2}\end{array}).$$

with each *V*_*j*_ being a 2 × 2 matrix, the entanglement of the mechanical modes *b*_1_ and *b*_2_ called logarithmic negativity can then be calculated^48,49^

25 $${E}_{N}=\,{\rm{\max }}\,\mathrm{[0},-\mathrm{ln}\,\mathrm{(2}\eta )]$$

with $$\eta ={2}^{-1/2}{\{\sum -{[{\sum }^{2}-4{\rm{\det }}({\sigma }_{{\rm{m}}})]}^{\mathrm{1/2}}\}}^{\mathrm{1/2}}$$ and $$\sum ={\rm{\det }}({V}_{1})+{\rm{\det }}({V}_{2})-2\,{\rm{\det }}({V}_{3})$$.

**Figure 3:**
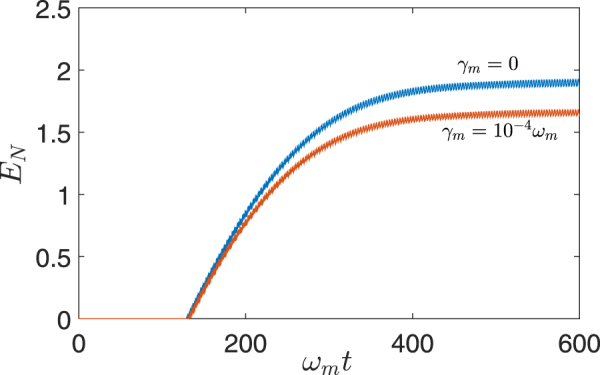
Time evolution of the mechanical entanglement evaluated using the full linearized Hamiltonian in Eq. (6) for two different values of the mechanical decay rate, with all mechanical and cavity modes are initially in thermal equilibrium with their baths. A set of experimentally feasible parameters^39,42,50,51^ are chosen here: *G*_+_/*G*_−_ = 0.8, ${\bar{n}}_{b}=2$, and (in units of *ω*_*m*_) *κ* = 0.1, *J* = 3, Δ_0_ = 4, *g* = 10^−5^, *G*_−_ = 0.03, *γ*_*m*_ = 0 (blue line), *γ*_*m*_ = 10^−4^ (orange line).

To demonstrate the mechanism of generating mechanical entanglement via cavity dissipation discussed priviously, we plot in Fig. 3 the time evolution of the entanglement *E*_*N*_ between two mechanical modes *b*_1_ and *b*_2_ with all mechanical and cavity modes initially in thermal equilibrium with their baths. The results are numerically evaluated with the full linearized Hamiltonian in Eq. (6), using a set of experimentally achievable parameters^39,42,50,51^. Obviously, there is no entanglement between *b*_1_ and *b*_2_ until the Bogoliubov modes *θ*_1_ and *θ*_2_ have been sufficiently cooled after some time. Then, following a dramatic increase, *E*_*N*_ eventually tends to be saturated with small vibrations which derive from the effects of non-resonant terms. The steady-state mechanical entanglement achieved (*E*_*N*_ ~ 1.6) in our scheme is much larger than the optomechanical entanglement (*E*_*N*_ ~ 0.63) generated in many previous schemes^1,4,21,36^ which are based on the coherent parametric interactions and subjected to the stability constraint. For larger mechanical decay rate, one has stronger interactions of the mechanical modes with the mechanical thermal baths, which raises the final effective temperature of Bogoliubov modes, accordingly reducing the steady-state mechanical entanglement as illustrated in Fig. 3.

As shown in related three-mode cases^30,33^, the amount of stationary entanglement is a nonmonotonic function of the ratio of the effective couplings *G*_+_/*G*_−_. The increase in *G*_+_ (holding *G*_−_ constant) has two competing effects. On the one hand, it can increase the squeezing parameter $r={\tanh }^{-1}({G}_{+}/{G}_{-})$ of the two-mode squeezed thermal states for the mechanical modes in the stationary regime, i.e., enhance the stationary entanglement. On the other hand, it will weaken the cooling effects of the Bogoliubov modes due to the declining coupling strength $\tilde{G}=\sqrt{{G}_{-}^{2}-{G}_{+}^{2}}$ between the cavity mode *a*_1(2)_ and the Bogoliubov mode *θ*_1(2)_. The achievable mechanical entanglement is determined by balancing these opposing effects.

Another effect of varying *G*_+_/*G*_−_ that needs particular attention is its influence on the dynamics of the mechanical mean values. All previous analyses are based on the assumptions that we have a stationary and well-behaved system dynamics which does not enter the strongly oscillating, unstable, or chaotic regime^52–56^. From Eqs (10), (12) and (14) we have

26 $$\frac{{G}_{+}}{{G}_{-}}=\frac{{E}_{2}[\kappa /2+i({{\rm{\Delta }}}_{0}+J-{\omega }_{1})]}{{E}_{1}[\kappa /2+i({{\rm{\Delta }}}_{0}+J-{\omega }_{2})]}.$$

**Figure 4:**
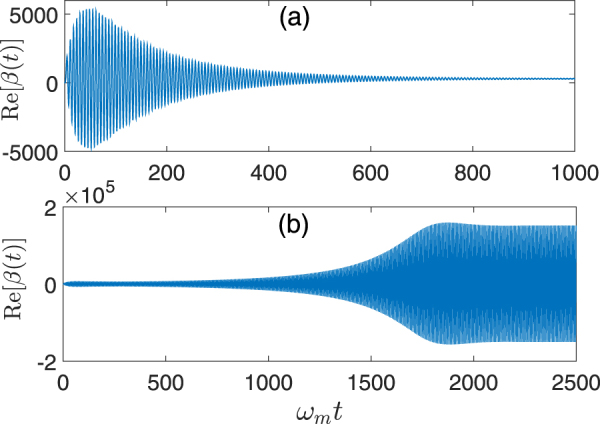
Dynamical behaviors of classical mechanical mean values numerically calculated from Eq. (4) with the mechanical decay rate *γ*_*m*_ = 10^−4^ *ω*_*m*_. (**a**) *G*_+_/*G*_−_ = 0.8 and (**b**) *G*_+_/*G*_−_ = 0.98. In both figures, all remaining parameters are the same as those in Fig. 3.

Assuming that all system parameters except the driving amplitudes are kept fixed, we have *G*_+_/*G*_−_ proportional to the ratio of the driving amplitudes *E*_2_/*E*_1_. Figure 4 shows the time evolution of the classical mechanical mean values *β*(*t*) for two selected values of *G*_+_/*G*_−_, which are numerically calculated via Eq. (4). After some transient time, *β*(*t*) reaches self-sustained oscillations. The oscillation amplitudes can be apparently different depending on the values of the ratio *G*_+_/*G*_−_. Generally, for those driving amplitudes *E*_2_/*E*_1_ corresponding to *G*_+_/*G*_−_ that are not very close to 1 (such as *G*_+_/*G*_−_ = 0.8), as demonstrated in Fig. 4(a) we have small mecahincal oscillations in the asymptotic regime so that $g[\beta (t)+\beta {(t)}^{\ast }]\ll {{\rm{\Delta }}}_{0}\sim {\omega }_{m}$ and ${\rm{\Delta }}(t)\simeq {{\rm{\Delta }}}_{0}$, ensuring that the effective Hamiltonian derived in Eq. (15) is valid. However, for some larger *E*_2_/*E*_1_ associated with *G*_+_/*G*_−_ only slightly less than 1 (such as *G*_+_/*G*_−_ = 0.98), *β*(*t*) oscillates with amplified amplitudes^55,56^ as shown in Fig. 4(b). In this case, the condition ${\rm{\Delta }}(t)\simeq {{\rm{\Delta }}}_{0}$ and accordingly the derived Hamiltonian in Eq. (15) for generating mechanical entanglement are no longer effective, thus no significant entanglement can be obtained.

Finally we would like to briefly discuss the experimental feasibility of our scheme. The microtoroidal cavities in our system shown in Fig. 1 can be fabricated at the edges of two separate chips. The whispering-gallery modes of the two microtoroidal cavities couple to mechanical modes via the radiation pressure, while the direct coupling between the two cavities can be achieved by placing them on nanopositioning systems capable of precisely controlling the distance^39,40^. An electro-optic modulator is exploited to generate the required time-modulated amplitudes of lasers pumping the whispering-gallery modes of the resonators by means of taper couplings. A set of optomechanical parameters comparable to the adopted ones in our scheme is reported in recent experiments with mechanical resonance frequency *ω*_*m*_ = 2*π* × 78 MHz, single-photon optomechanical coupling rate *g* = 4.35 × 10^−5^ *ω*_*m*_, cavity decay rate *κ* = 0.09 *ω*_*m*_, mechanical decay rate *γ*_*m*_ = 1.5 × 10^−4^ *ω*_*m*_, and thermal occupancy ~2 using cooling technique^42,50^. Although we use coupled microtoroidal optomechanical system in our scheme, the mechanism for generating distant mechanical entanglement can be extended to other coupled optomechanical systems, such as microwave-circuit optomechanical systems where similar parameters are achieved in recent state-of-the-art experiments^57,58^.

## Conclusion

In summary, we have proposed a scheme to generate steady-state mechanical entanglement in a coupled optomechanical system. By applying four-tone driving lasers with weighted amplitudes and specific frequencies, we can get beam-splitter-like interactions between the cavity modes and the delocalized Bogoliubov modes of the two mechanical oscillators, which enables the cooling of the Bogoliubov modes by the cavity decay as long as the mechanical decay rate is small. The mechanical oscillators are then driven to close to two-mode squeezed states when the Bogoliubov modes have been sufficiently cooled to near vacuum after some time of dissipative dynamics. By balancing the opposing effects of varying the ratio of the effective optomechanical couplings and carefully avoiding the system parameters that may lead to the unwanted amplified oscillations, we obtain steady-state mechanical entanglement that is significantly larger than the generated entanglement based on the coherent parametric interactions in many previous schemes.
